# Potential role of endocrine gastrin in the colonic adenoma carcinoma sequence

**DOI:** 10.1038/sj.bjc.6600509

**Published:** 2002-08-27

**Authors:** S A Watson, T M Morris, D F McWilliams, J Harris, S Evans, A Smith, P A Clarke

**Affiliations:** Academic Unit of Cancer Studies, University of Nottingham, Nottingham, NG7 2UH, UK

**Keywords:** gastrin, CCK-2 receptors, colon adenomas, APC^*Min*^ mouse

## Abstract

The role of hyper-gastrinaemia in the incidence of colonic cancer remains to be clarified. The aim of this study was to determine whether cholecystokinin-2 (CCK-2) receptor expression predicts the sensitivity of human colonic adenomas to the proliferative effects of serum hyper-gastrinaemia. Gene expression of the classical (74 kDa) CCK-2 receptor in human colonic adenoma specimens and cell lines, was quantified by real-time PCR. Western blotting, using a CCK-2 receptor antiserum, confirmed protein expression. A transformed human colonic adenoma was grown in SCID mice, with hyper-gastrinaemia induced by protein pump inhibitors. CCK-2 receptor blockade was achieved by using neutralising antiserum. Both human colonic adenoma cell lines and biopsies expressed CCK-2 receptor mRNA at levels comparable with CCK-2 receptor transfected fibroblasts and oxyntic mucosa. Western blotting confirmed immunoreactive CCK-2 receptor bands localised to 45, 74 and 82.5 kDa. Omeprazole and lansoprazole-induced hyper-gastrinaemia (resulting in serum gastrin levels of 34.0 and 153.0 pM, respectively) significantly increased the weight of the human adenoma grafts (43% (*P*=0.016) and 70% (*P*=0.014), respectively). The effect of hypergastrinaemia on tumour growth was reversed by use of antiserum directed against the CCK-2 receptor. Hyper-gastrinaemia may promote proliferation of human colonic adenomas that express CCK-2 receptor isoforms.

*British Journal of Cancer* (2002) **87**, 567–573. doi:10.1038/sj.bjc.6600509 www.bjcancer.com

© 2002 Cancer Research UK

## 

Gastrin peptides have been shown to be growth factors for normal colonic epithelial cells (Sobhani *et al*, 1993; Koh *et al*, 1997; Renga *et al*, 1997), as well as those that have undergone malignant transformation (Winsett *et al*, 1986; Watson *et al*, 1989). Such findings may be of clinical significance as raised serum gastrin levels may be a common occurrence, being a side effect following either infection with *Helicobacter pylori* (HP) (Smith *et al*, 1990; Graham *et al*, 1991) or administration of proton pump inhibitors (Festen *et al*, 1986; Lind *et al*, 1988). The ability of elevated serum gastrin levels to promote colorectal carcinogenesis has been evaluated in a number of chemically induced *in vivo* models. Omeprazole-induced hyper-gastrinaemia has consistently failed to increase tumour incidence in carcinogen-induced colorectal malignancy in rats (Penman *et al*, 1993; Hurwitz *et al*, 1995; Pinson *et al*, 1995). Conversely, surgical induction of hyper-gastrinaemia has resulted in an increased incidence of chemically-induced tumours in two studies (Mcgregor *et al*, 1982, Karlin *et al*, 1985). Furthermore, hyper-gastrinaemia decreased survival in the *APC^Min^* mouse model of intestinal polyposis (Watson and Smith, 2001). The reasons for such divergent results are unclear but they highlight the need to examine the risk of hyper-gastrinaemia in more clinically relevant models.

The majority of epidemiological studies have so far failed to define the true risk of hyper-gastrinaemia as most have not controlled for confounding factors, known to elevate serum gastrin, in the control population (Elsborg and Mosbech, 1979; Brinton *et al*, 1989; Orbuch *et al*, 1996). A well-controlled clinical study by Thorburn *et al* (1998) showed that serum hyper-gastrinaemia was associated with a 3.3-fold increase in the relative risk of developing colorectal cancer with 8.9% of colorectal cancers attributable to increased serum gastrin levels.

The cholecystokinin-B (CCK-2) receptor mediates the physiological effects of gastrin, including growth promoting effects on pre-malignant and malignant colonic mucosa (Smith and Watson, 2000). Furthermore, gastrin stimulation of CCK-2 receptors expressed by AR42J cells was shown in an *in vitro* system to reverse apoptosis induced by serum withdrawal through the induction/activation of protein kinase BAkt (Todisco *et al*, 2001). A number of isoforms of the CCK-2 receptor have now been discovered (Song *et al*, 1993; Miyake, 1995; Ohtaki *et al*, 1996). These include the ‘long isoform’ which has, due to alternate splicing, an additional five amino acids located within the third intracellular domain (Song *et al*, 1993; Ohtaki *et al*, 1996). Use of an alternative first exon, 1b, which remains untranslated, leads to a receptor truncated at the amino-terminus, known as the ΔCCK-2. This receptor exists on human colorectal adenocarcinoma specimens and cell lines which have an activated gastrin gene, and therefore has been implicated in mediating the autocrine pathway (Mcwilliams *et al*, 1998). More recently, CCK-2 receptors with retained introns 2 and 4 have been described in malignant pancreatic and colorectal cells, respectively (Goetze *et al*, 2000, Hellmich *et al*, 2000) with the intron 4 retaining CCK-2 receptor inducing ligand-independent constitutive intra-cellular Ca^2+^ mobilisation (Hellmich *et al*, 2000). However their relevance regarding colonic tumour proliferation progression remains to be clarified.

As the classical CCK-2 receptor mediates the proliferative response to endocrine gastrin, the aim of the present study was to define the significance of non-truncated CCK-2 receptor isoforms expressed by human colorectal adenomas.

## MATERIALS AND METHODS

### Human colonic adenoma cell lines

Three human adenoma cell lines were examined in this study; AA/C1 and RGC2/42 are non-tumorigenic adenoma cell lines and AA/C1/SB/10C is a semi-transformed variant of AA/C1 with limited *in vivo* tumorigenic potential (Williams *et al*, 1990). A table of known mutations is shown in Table 1

**Table 1:**
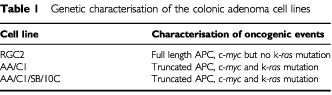
Genetic characterisation of the colonic adenoma cell lines

. These cells were kindly provided by Professor C Parasceva, CRC Laboratories, University of Bristol, UK. The cell lines were maintained at 37°C in humidified conditions with 5% CO_2_, in conditioned medium from mouse NIH3T3 fibroblasts prepared in DMEM tissue culture medium (GIBCO, Irvine, Scotland) containing 20% heat-inactivated foetal calf serum (FCS, Sigma, Poole, UK), 2 mM L-glutamine (Sigma), insulin (0.2 Units ml^−1^, Sigma) and hydrocortisone (1 μg ml^−1^, Sigma).

The human colorectal adenocarcinoma cells used were LoVo and HT29, both obtained from the ECACC (Porton Down, UK), and the human liver-invasive colorectal adenocarcinoma, C170HM_2_, established within the Cancer Studies Unit, University of Nottingham, UK (Watson *et al*, 1993). The CCK-2 receptor transfected NIH3T3 cells were a kind gift from Professor Matsui, Kobe University, Japan, as previously described (Ito *et al*, 1993). All the above cell lines were maintained in RPMI tissue culture medium supplemented with 10% FCS and 2 mM L-glutamine.

### Growth in severe combined immuno-deficient (SCID) mice

AA/C1/SB/10C cells were injected subcutaneously (s.c.) into the left flank of mixed sex SCID mice (age 4–5 weeks, bred within the Cancer Studies Unit, University of Nottingham, UK) at a cell concentration of 2×10^6^ in 100 μl. After growth of a palpable tumour (approximately 3 months), tumour tissue was pooled from donor mice and 3 mm^3^ grafts transplanted s.c. into experimental SCID mice anaesthetised with Hypnorm (0.315 ng ml^−1^, Jannsen, Belgium) and Hypnovel (5 ng ml^−1^, Roche, Switzerland). Tumour grafts were measured as previously described (Watson *et al*, 1993). One hour before study termination, mice were injected with 10 mg kg^−1^ bromodeoxyuridine (BrdU) into the peritoneal cavity. At the termination of the study, tumour tissue was taken and fixed in formal calcium (4% formaldehyde, 10% CaCl_2_ in distilled water) for immunohistological evaluation. Tumour tissue was snap-frozen in liquid N_2_ for molecular biology studies. UK Co-ordinating Committee for Cancer Research (UKCCCR) Guidelines were adhered to throughout all animal experimentation.

### *In vivo* treatment

Omeprazole (Hospital Pharmacy, Queen's Medical Centre, Nottingham, UK) was administered at a dose of 75 mg kg^−1^ by gavage as previously described (Watson and Smith, 2001). Lansoprazole (Hospital Pharmacy as above) was prepared in sterile distilled H_2_O and administered orally, daily, in 100 μl volumes at a dose of 25 mg kg^−1^.

Affinity purified polyclonal CCK-2 receptor (gastrin receptor epitope (GRE)-1, Watson *et al*, 1998) antiserum (Aphton Corporation, CA, USA) was administered at a dose of 100 μg protein in a 200 μl volume, i.v., daily. This antiserum has been shown to inhibit ligand binding and intra-cellular signalling of the CCK-2 receptor (Watson *et al*, 2000; Mcwilliams *et al*, 2001). Protein G purified normal rabbit serum (Aphton, CA, USA), at the same protein concentration, was administered to control mice.

Amidated serum gastrin levels were measured by Professor Andrea Varro (Physiology Department, University of Liverpool) using the L2 antiserum, as previously described (Nemeth *et al*, 1993).

### Measurement of the proliferation index of the adenoma tissue

Following formalin fixation, specimens were embedded in paraffin, 4 μM sections were cut by a microtome and stained with an antibody directed against BrdU as previously described (Watson and Smith, 2001). Nuclei were selected on the basis of colour and were expressed as a percentage of the total adenoma tissue in *n*=20 high power fields. Inter-observer variation was found to be 7.5% and intra-observer variation 8.8%. Image analysis was performed by the use of the Leica Qwin image processing system (Leica Microsystems, Cambridge, UK).

### Human specimens

Either paraffin-embedded or snap frozen human colonic normal mucosa (from patients without a malignancy), mucosa from the resection margin of patients with an adenocarcinoma and villous adenoma and adenocarcinoma specimens were used for the real-time screen of CCK-2 receptor expression.

For the Western blotting studies, snap frozen tissue was obtained from resection margin mucosa samples, villous adenomas and adenocarcinomas, prospectively. The adenomas were mainly synchronous to adenocarcinomas. Real-time PCR was performed on respective samples, in parallel.

### Extraction of RNA

For frozen and paraffin-embedded tissue, total RNA was extracted as previously described (Mcwilliams *et al*, 1998; Smith and Watson, 2000) and reverse transcription performed (Mcwilliams *et al*, 1998).

Primer pairs, for use in real time PCR, were designed to amplify only the human classical CCK-2 receptor. The sequences are shown in Table 2

**Table 2:**
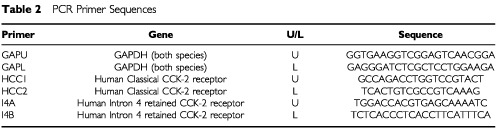
PCR Primer Sequences

.

### Real Time PCR to quantify CCK-2 receptor expression

RNA was reverse transcribed from random hexamer primers (Pharmacia) using Superscript RT (GibcoBrl, UK). Real Time PCR was performed using the 5700 Sequence Detection System (PE Applied Biosystems, Warrington, UK). Each PCR was performed according to manufacturer's instructions using 1 μl of sample complementary DNA (cDNA) in a 25 μl reaction volume. The reaction buffer was prepared from the SYBR Green PCR Core Kit (PE Applied Biosystems) and consisted of 1×SYBR Green PCR Buffer (PE Applied Biosystems), 3 mM MgCl_2_, 0.2 mM dATP, 0.2 mM dCTP, 0.2 mM dGTP, 0.4 mM dUTP, 0.25 U AmpErase UNG (PE Applied Biosystems) and 0.625 U Amplitaq Gold (PE Applied Biosystems). The CCK-2 receptor primers and the primers for the house-keeping gene, glyceraldehyde-3-phosphate-dehydrogenase (GAPDH) were included in parallel reactions at a final concentration of 100 nM each. Negative controls were included in which the reverse transcriptase was omitted, to control for genomic DNA contamination.

The fluorescence of the SYBR green dye bound to the CCK-2 receptor and GAPDH PCR products was measured, after each cycle, by the 5700 System and the cycle number was recorded when the accumulated signal crossed an arbitrary cycle threshold (Ct). The relative gene expression for each sample was determined using the formula 2^ct(GAPDH)−ct(CCK−2)^, in which CCK-2 receptor gene expression was normalised to GAPDH levels. CCK-2 receptor mRNA levels of the human adenoma xenografts were measured by the ELOCA assay, as previously described (Watson *et al*, 1999a). Each sample (*n*=5) was repeated 2–3 times.

### Western blotting

Western blotting was performed on cell pellets or human tissue, snap frozen in liquid nitrogen, using a rabbit polyclonal antiserum directed against the amino terminus of the classical human CCK-2 receptor, as previously described (Watson *et al*, 1998).

### Statistical analysis

Results were assessed using the Minitab statistical programme for the PC by the Mann–Whitney U non-parametric test (real time PCR results) and the *in vivo* data by Student's *t*-test or one-way analysis of variance.

## RESULTS

### Real time CCK-2 receptor profile of colonic adenoma and adenocarcinoma cell lines

**Figure 1:**
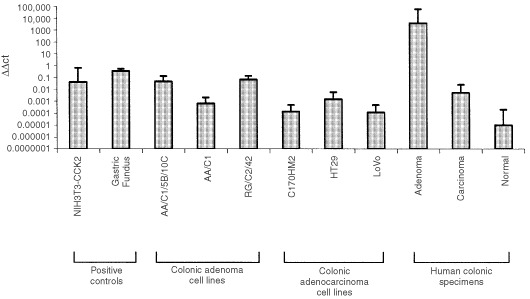
Gene expression of CCK-2 wild type (74 kDa) receptors in a panel of colonic adenoma and adenocarcinoma specimens and cell lines as determined by real time PCR. Mean expression of 2 to 3 separate PCR reactions per sample are shown together with the standard deviations of the mean.

The mean ΔΔCt (relative gene expression of CCK-2 receptor normalised to GAPDH) of each cell line examined (means of 2–3 repeat PCR reactions performed on two separate cDNA preparations) is shown in Figure 1

. The positive control cells used were NIH3T3s transfected with the human classical CCK-2 receptor and the negative control was the human fibroblast cell line BRIG1, which showed no receptor expression at the detection sensitivity of the real time PCR assay (ΔΔCt<1×10^−12^ in five repeated assays, results not shown).

The three human adenoma cell lines expressed classical CCK-2 receptor mRNA. The ΔΔCt levels of CCK-2 receptor mRNA in the two non-transformed adenoma cell lines were significantly greater when compared to the transformed adenoma, AA/C1/5B/10C (*P*=0.0014, AA/C1 and *P*=0.0081, RGC2/42, Mann–Whitney). The ΔΔCt levels for the receptor were also higher in AA/C1 and RGC2/42 cells when compared to the adenocarcinoma cell lines (*P*=0.0008, *P*=0.0032 respectively). However, CCK-2 receptor ΔΔCt levels of the two non-transformed adenomas were not significantly different from those expressed by the NIH3T3 CCK-2 receptor transfectants and were lower than the levels expressed by oxyntic mucosa (*P*=0.0108, for AA/C1 and *P*=0.0081 for RGC2/42). When comparing the adenocarcinoma cell lines, there was no significant difference in ΔΔCt for the classical CCK-2 receptor between all three lines, however, the ΔΔCt values were lower than the level expressed by the NIH3T3 receptor transfectant (*P*=0.0107), and oxyntic mucosa (*P*=0.001).

### Real time CCK-2 receptor profile of human colonic normal mucosa, adenoma and adenocarcinoma specimens

As real-time quantification was to be performed, mRNA extracted from surgically resected specimens was assessed for high integrity by careful analysis of house-keeping gene expression. Maximal expression of a panel of house-keeping genes (glyceraldehyde phosphate dehydrogenase (GAPDH), Glycerol-3-phosphate dehydrogenase (G3PDH), β-actin, 18S RNA) has previously been validated by the use of human colonic tissue samples immediately snap-frozen at endoscopy (D McWilliams, personal communication). Only samples with the correct house-keeping mRNA profile were used, resulting in limited numbers for the final assessments, as follows: (i) non-malignant colonic mucosa (*n*=5), (ii) resection margin mucosa from patients with a colonic adenocarcinoma (*n*=12), (iii) villous adenomas (*n*=11), and (i.v.) colonic adenocarcinomas (*n*=13). The adenoma specimens expressed greater CCK-2 receptor ΔΔCts than the adenocarcinomas (*P*<0.0001, Mann–Whitney), resection margin normal mucosa (*P*=0.0003), NIH3T3 CCK-2 receptor transfectants (*P*=0.005) and oxyntic mucosa (*P*=0.0001). The adenocarcinomas expressed lower CCK-2 receptor ΔΔCt levels than oxyntic mucosa (*P*=0.0001) and NIH3T3 CCK-2 receptor transfectants (*P*=0.018), but higher levels than resection margin mucosa (*P*=0.0063). Normal colonic mucosa taken from patients without a malignancy showed no receptor expression at the detection level of the real time PCR assay (ΔΔCt <1×10^−12^ for all samples examined, data not shown).

### Western blot analysis of CCK-2 receptor expression of human adenoma cell lines and colonic normal resection margin mucosa, adenoma and adenocarcinoma specimens

**Figure 2:**
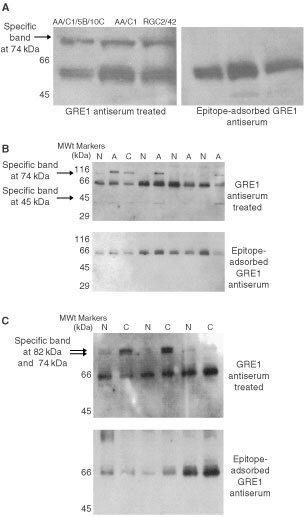
Western blot analysis showing CCK-2 receptor isoform immuno-reactivity of human colonic adenoma specimens and human colonic adenoma cell lines. (**A**) Human colonic adenoma cell lines. (**B**) Human colonic adenoma specimens. Resection margin normal (N), adenoma (A) and adenocarcinoma (C) specimens from individual patients. (**C**) Human colonic adenocarcinoma specimens. Resection margin normal (N) and adenocarcinoma (C) specimens from individual patients.

Using the GRE1 antiserum, an immunoreactive band at the 74 kDa molecular weight was expressed by the human adenoma cell lines (Figure 2A

) and the human colonic adenoma specimens (Figure 2B shows a panel of representative specimens from a series of eight so far examined), together with normal resection margin colonic mucosa and in one example, a synchronous adenocarcinoma. A specific immunoreactive band was also shown at 45 kDa. The 66 kDa band was non-specific as it was also expressed in the negative control blots (epitope adsorbed antiserum).

The human colonic adenocarcinomas (a representative panel from a series of >30 screened) all expressed an immunoreactive doublet at both 74 and 82 kDa following staining with the GRE1 antiserum (Figure 2C). No 45 kDa band was present. The resection margin normal mucosa expressed weaker bands, the doublet being evident in 1 out of 3 samples shown and 74 kDa bond expressed exclusively in 1 out of 3. The GRE1 Western blot profiles of the colon adenocarcinoma cell lines have previously been published (Watson *et al*, 1998). Real-time PCR confirmed gene expression of the CCK-2 receptor in selected samples (data not shown).

### *In vivo* effect of hyper-gastrinaemia on the growth ofAA/C1/SB/10C

The mean serum amidated gastrin levels in the vehicle treated mice (*n*=7/8 replicates/group) was 14.0 pM (standard deviation of 2.4) compared to 34.0 pM in the omeprazole treated mice (standard deviation of 10.3).

The terminal mean tumour cross-sectional areas and weights from two separate studies are shown in Table 3

**Table 3:**
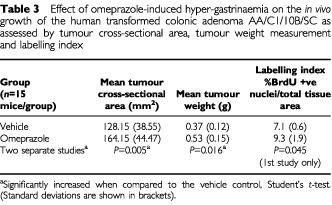
Effect of omeprazole-induced hyper-gastrinaemia on the *in vivo* growth of the human transformed colonic adenoma AA/C1/10B/SC as assessed by tumour cross-sectional area, tumour weight measurement and labelling index

. Omeprazole induced a 28% increase in tumour area (*P*=0.005, Student's *t*-test) and a 43% increase in tumour weight (*P*=0.016). The BrdU labelling index of tumours treated with omeprazole was assessed in the first study only and there was a 1.3-fold increase over vehicle treated controls (*P*=0.045).

### CCK-2 receptor mRNA expression in the adenoma xenografts

CCK-2 receptor mRNA was measured in adenoma xenografts from both vehicle control and omeprazole treated mice (Table 4

**Table 4:**
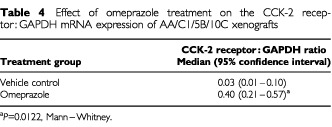
Effect of omeprazole treatment on the CCK-2 receptor : GAPDH mRNA expression of AA/C1/5B/10C xenografts

). There was a 13-fold elevation in the CCK-2 receptor : GAPDH ratio in omeprazole treated tumours (*P*=0.0122, Mann–Whitney).

### Western blotting of the adenoma xenografts

**Figure 3:**
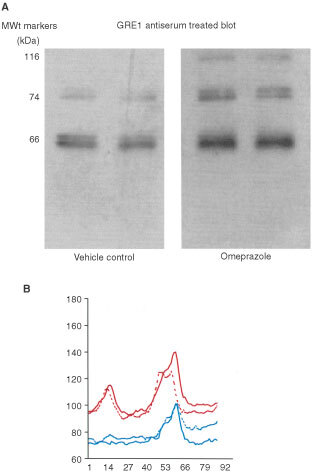
Western blot analysis showing CCK-2 receptor immuno-reactivity of human colonic adenoma xenografts. (**A**) Western blots (±omeprazole treatment). (**B**) Densitometry scans of the immunoreactive bands generated from the Western blots. Red line: imunoreactive bands from grafts obtained from omeprazole-treated mice. Blue line: immuno reactive bands from grafts obtained from vehicle-treated mice.

Western blotting was performed on four xenografts from each experimental group. Each sample was a pool of two xenografts and each was loaded onto the gel at an equivalent protein concentration. The results are shown in Figure 3

. There was specific immunoreactivity at 74, 82.5 and 120 kDa (blot shown in Figure 3a). The latter two bands were only visible in adenomas treated with omeprazole. Densitometry scans were generated from the Western blots and the specific bands are shown in Figure 3b. The scans highlight the increase in density of the 74 and 82.5 kDa doublet in adenomas from omeprazole treated mice and the novel expression of higher molecular weight immunoreactivity at 120 kDa.

### Reversal by blockade of the CCK-2 receptor

**Figure 4:**
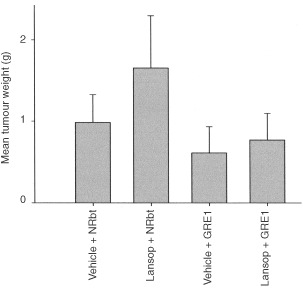
Final adenoma weights of mice treated with lansoprazole to generate hyper-gastrinaemia and co-administered with a polyclonal affinity purified antiserum, GRE1, directed against the CCK-2 receptor. Group 1: Oral vehicle (phosphate buffered saline (PBS), pH 7.2) + Protein A purified normal rabbit serum (*n*=11 mice). Group 2: Lansoprazole (orally dosed daily, 0.75 mg/mouse) + normal rabbit serum (*n*=10 mice). Group 3: Oral vehicle + GRE1 antiserum (*n*=12 mice). Group 4: Lansoprazole + GRE1 antiserum (*n*=11 mice).

To confirm that the *in vivo* growth effect was directly related to serum hypergastrinaemia, lansoprazole was used to generate both greater increases in serum gastrin (mean of 153.0, s.d. of 49.0 pM) and to dissociate any molecule-specific effects of omeprazole. GRE1 antiserum was co-administered to reverse the growth effects by receptor blockade. Figure 4

shows that lansoprazole increased the final mean tumour weight by 70% (*P*=0.014, one way analysis of variance). This was reversed by co-administration of GRE1 antiserum such that it was non-significant from the vehicle control (*P*=0.148 when compared to the vehicle control; *P*=0.001 when compared to lansoprazole + normal rabbit serum). GRE1 antiserum alone, significantly reduced basal growth by 38% (*P*=0.014).

### Intron IV CCK-2 receptor gene expression by theAA/C1/SB/10C adenoma cell line

Real time PCR on the above cell line confirmed gene expression of the CCK-2 receptor retaining intron IV. The mean ΔΔCt of two separate cDNA samples performed twice was 0.021 (standard deviation of 0.014) indicating positive expression.

## DISCUSSION

Serum hypergastrinaemia has become a more common occurrence in recent years, partly as a result of an increase in the use of proton pump inhibitors for acid secretory disorders (Festen *et al*, 1986; Lind *et al*, 1988). Furthermore, a positive temporal relationship has been observed between hyper-gastrinaemia and increased incidence of colon cancer with 8.9% of colon cancer being shown to be attributable to elevated gastrin levels (Thorburn *et al*, 1998), indicating a sub-group of patients exhibiting increased sensitivity.

Recent publications have indicated that for serum hyper-gastrinaemia to impact upon tumour progression there needs to be co-expression of CCK-2 receptors. In transgenic mouse models of hypergastrinaemia, gastrin induces CCK-2 receptor expression in gastric pit cell precursors (Nakajima *et al*, 2002) and invasive gastric carcinoma (Wang and Dockray, 1999). To induce pancreatic carcinomas, the hyper-gastrinaemic hGAS transgenic mouse model was crossed with the ELAS CCK-2 receptor mouse which over-expresses CCK-2 receptors in the pancreas. In 13% of the hybrid mice, carcinoma of the pancreas was present by 18 months (Clerc *et al*, 2002).

Studies determining the effect of hyper-gastrinaemia on pre-malignant polyps in the human colon have been limited despite it being shown that CCK-2 receptors are expressed by human adenomas (Smith and Watson, 2000). The incidence of colonic polyps was reported to be increased in patients with *Helicobacter pylori* infection (Breuer-Katschinski *et al*, 1999) but any relationship between serum hyper-gastrinaemia and adenoma/polyp proliferation remains tenuous. In the present study, maximal expression of classical CCK-2 receptor gene was shown in both human colonic adenoma cell lines and human adenoma specimens when compared to colonic adenocarcinomas. This is the first study to indicate that the proliferative effects of serum hyper-gastrinaemia on tumour progression may have maximum impact during the adenoma stage. It may also explain the sensitivity of the APC^*Min*^ mouse to elevated gastrin levels, which resulted in decreased survival (Watson and Smith, 2001). The 8.9% colorectal cancers arising from serum hyper-gastrinaemia may therefore represent a population of patients with pre-existing colonic polyps.

The reason for the lower gene expression of the classical CCK-2 receptor at the adenocarcinoma stage is unknown. At the protein level we have previously shown that the intron IV retained CCK-2 receptor splice variant may be the predominant form expressed by colonic adenocarcinomas (Clarke *et al*, 1999; 2001). In the present study, an 82 kDa immuno-reactive band was co-expressed with the 74 kDa band (relating to the molecular weight of the classical CCK-2 receptor) following Western blotting with a CCK-2 receptor antiserum in a series of human colonic adenocarcinomas. The larger molecular weight isoform of the CCK-2 receptor was absent from a panel of human adenomas. The 82 kDa band relates (in terms of molecular weight) to the intron IV CCK-2 receptor isoform that would be detected by the antiserum as it is directed against the amino-terminal domain common to both CCK-2 receptor isoforms.

The primers used in the real time PCR method were designed for optimal detection of classical CCK-2 receptor and would detect the intron IV containing receptor much less efficiently. Thus, the variations in CCK-2 receptor gene expression between adenomas and adenocarcinomas could reflect a shift to greater expression of intron IV retained CCK-2 receptor isoforms in adenocarcinomas.

Alternatively, it is known that gastrin gene expression is greater in colonic adenocarcinomas than in adenomas (Watson *et al*, 1999b) and in a series of GI cell lines transfected to over-express the gastrin gene, there was down-regulation of gene and protein expression of classical CCK-2, but not Intron IV CCK-2, receptor isoforms (S Evans, personal communication). Therefore, increased gastrin gene expression could be partly responsible for the down-regulation in CCK-2 receptor gene expression seen in the adenocarcinomas. Expression of CCK-2 receptors by human colonic adenomas suggests that the cells may be responsive to serum hyper-gastrinaemia. The CCK-2 receptors expressed by AA/C1/1B/5B were shown to be functional, as following *in vivo* growth, the receptors were up-regulated at both the gene and protein level and mediated a growth response to increased serum gastrin levels induced by administration of protein pump inhibitors. The significant but modest increase in BrdU uptake in omeprazole treated mice may reflect the poorly vascularised status of the subcutaneous grafts at the end-stage of the study. Tumour growth in the lansoprazole treated mice was greater than in the omeprazole treated mice, which may relate to the higher serum gastrin levels, and was completely reversed by co-administration of antiserum blockading the CCK-2 receptor, confirming the growth effect as gastrin-specific.

Interestingly, following growth in hyper-gastrinaemic mice, *de novo* expression of the 82 kDa form of the CCK-2 receptor was confirmed in AA/C1/SB/10C grafts. This may indicate a shift to a more malignant phenotype in the present adenoma model following exposure to gastrin. Furthermore, a CCK-2 receptor isoform of molecular weight 120 kDa was exclusively expressed in adenoma grafts from hyper-gastrinaemic mice, the structure/function of which has so far not been described.

Thus further clinical studies are warranted to examine the proliferation of polyps from patients with elevated levels of serum gastrin. It may be that the 8.9% of total colorectal cancers that are attributable to serum gastrin levels may arise in patients with undiagnosed colonic polyps, due to the increased expression of CCK-2 receptors on these pre-malignant lesions. Carefully controlled epidemiological studies are necessary to relate hyper-gastrinaemia to the incidence of colorectal cancer, considering the widespread use of protein pump inhibitors.
